# D81 Mutants Reveal Hidden EF-Tu Diversity while Natural Sequences Preserve Aspartate

**DOI:** 10.34133/csbj.0167

**Published:** 2026-07-10

**Authors:** Jordan L. Johnson, Yuhong Wang

**Affiliations:** ^1^ Department of Biology and Biochemistry, University of Houston, Houston, TX 77204, USA.; ^2^ Department of Chemistry, University of Houston, Houston, TX 77204, USA.

## Abstract

Conformational dynamics are central to the function of many proteins, including the translational guanosine triphosphatase EF-Tu. AlphaFold2-based approaches such as AF-Cluster use sequence ensembles to sample alternative conformations, but most clustering analyses focus on predicted structures without examining the sequence and phylogenetic or ecological context of the multiple sequence alignments (MSAs) that generate them. Complex landscapes can also be poorly resolved by single-step clustering, because one global metric may conflate motions occurring at different architectural levels. Here, we address these limitations using a structure-guided strategy for EF-Tu. Query MSAs were clustered to generate diverse predicted structures, then sorted in 2 steps: first by the interdomain orientation of helix 183 to 199, capturing global open and closed forms, and then by switch I loop 54 to 58, resolving local variation at the guanosine triphosphate binding pocket. Comparing wild-type and D81 mutant queries showed that D81 substitution reshapes sequence retrieval and recruits a broader ecological range of organisms. Although D81A, D81F, and D81K retained ribosome-stimulated guanosine triphosphatase activity under the tested conditions, position 81 remained aspartate across virtually all retrieved sequences, indicating experimental substitutability but evolutionary invariance. AlphaFold3 modeling with ribosomal RNA components further suggested weakened Mg^2+^ coordination in the guanosine diphosphate state and guanosine triphosphate-specific sarcin–ricin loop displacement, consistent with possible effects on nucleotide handling or downstream coupling rather than loss of hydrolysis. These findings establish mutant-seeded MSA perturbation as a strategy for uncovering hidden sequence, ecological, and conformational diversity in conserved proteins.

## Introduction

Conformational landscapes are central to many protein functions, including translational guanosine triphosphatases (GTPases) [1]. However, experimental structure determination often captures low-energy endpoint states, leaving intermediate conformations difficult to resolve. AlphaFold, RoseTTAFold, and related structure prediction tools have begun to fill this gap, and methods such as AF-Cluster, multiple sequence alignment (MSA) subsampling, and SPEACH AF show that selected MSA inputs can drive prediction of alternative conformations [2–10]. However, these approaches often treat the MSA mainly as a folding tool, without analyzing the sequence and phylogenetic or ecological context behind each predicted conformation. We recently began bridging this gap by combining AlphaFold prediction with ancestral and phylogenetic analysis of elongation factor G [11]. In that work, we noticed a limitation shared by existing conformational-sampling methods: A single global structural coordinate cannot reliably sort predicted structures, because structures differing at different regions can yield the same root mean square deviation (RMSD) to a common reference yet represent very different conformations. Existing approaches do not resolve this degeneracy, and in our earlier work, we addressed it by selecting multiple reference structures through visual inspection. Here, we extend that direction with a systematic, structure-guided framework that links conformational clusters to their sequence and ecological origins and developed the hierarchic 2-step structure clustering strategy on *Escherichia coli* EF-Tu protein.

As a translational GTPase, EF-Tu delivers aminoacyl transfer RNA (tRNA) to the ribosome as part of the ternary complex with guanosine triphosphate (GTP) and couples codon recognition to GTP hydrolysis and tRNA accommodation [12,13]. This process requires allosteric communication from the codon–anticodon duplex to the GTP binding pocket, which adopts switch-open and switch-close states, and onward to the global rearrangement of domain I relative to domains II to III, defined here as D-open and D-close states (Fig. S1) [14]. Cognate tRNA strongly stimulates GTP hydrolysis, promoting D-open conformation to release the tRNA into the ribosomal A site [15,16]. However, the GTP binding pocket state does not strictly track nucleotide identity, nor does it map cleanly onto the global D-open/close states. For example, EF-Tu bound to a GTP analog has been observed in a D-open conformation equivalent to the guanosine diphosphate (GDP)-bound form, indicating that local pocket, and global domain conformations can vary without strict coupling [17,18]. EF-Tu also has moonlighting functions beyond canonical elongation, including roles in ribosome hibernation, cell communication, chaperone activity, and bacterial pathogenesis, each of which may impose distinct conformational and sequence constraints [19–22]. Therefore, the loose coupling among nucleotide state, local switch conformation, and global domain rearrangement may be functionally important, but the sequence and evolutionary constraints shaping these states remain unclear.

**Fig. 1.**
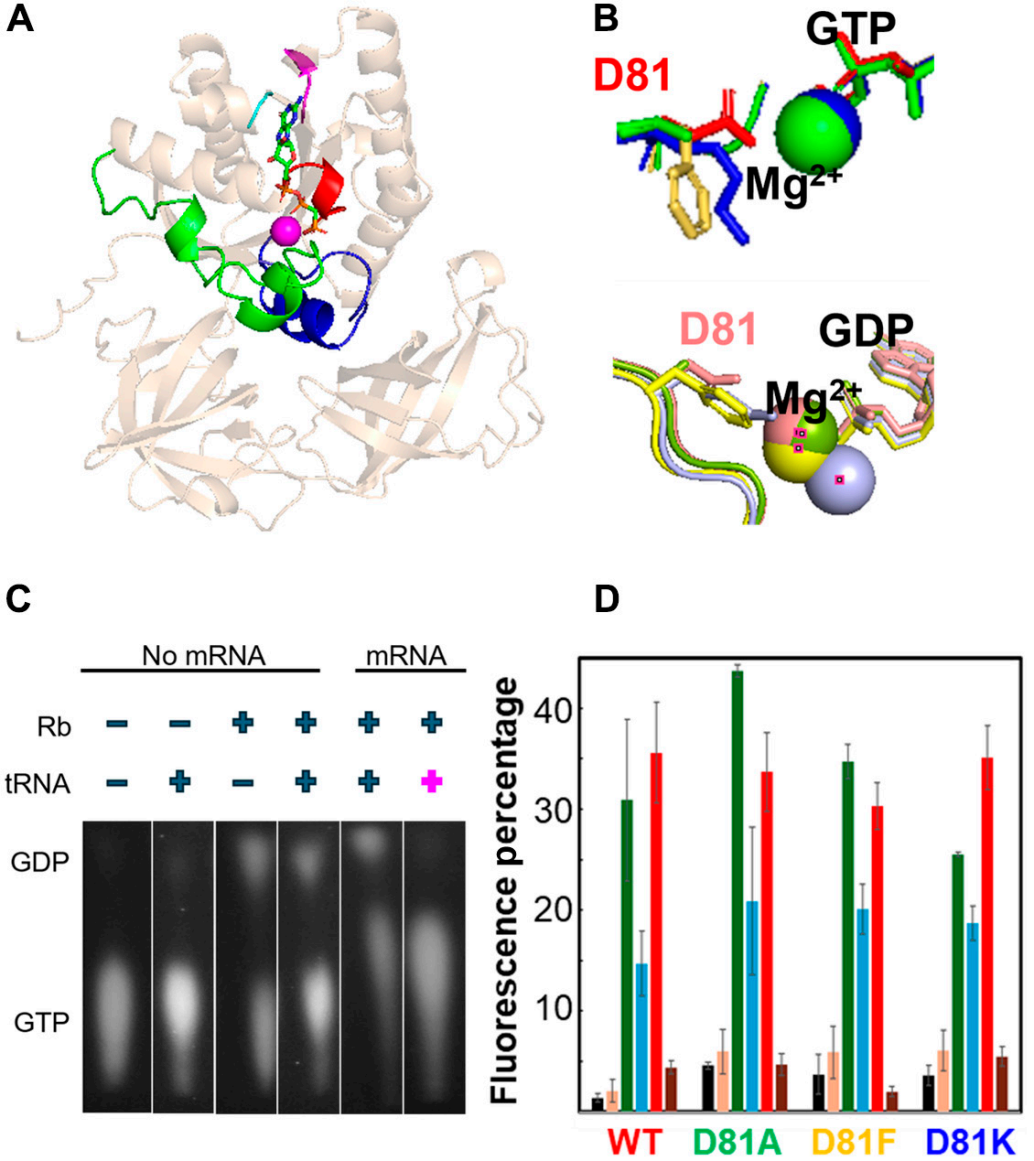
Guanosine triphosphate (GTP) hydrolysis center of EF-Tu. (A) The 5 conserved motifs involved in nucleotide coordination within the GTP binding pocket: P-loop (red, residues 19 to 26), switch I (green, residues 41 to 63), switch II (blue, only 81 to 91 shown), G4 (magenta, residues 136 to 139), and G5 (cyan, residues 174 to 177). Circle indicates Mg^2+^. (B) AlphaFold3 predicted conformations of D81 and its mutants in GTP- and guanosine diphosphate (GDP)-bound states. (C) Representative thin-layer chromatography (TLC) images of mant-GTP hydrolysis by EF-Tu. Magenta cross indicates near-cognate tRNA. Rb, ribosome. (D) Bar plot summarizing guanosine triphosphatase (GTPase) activity across 6 conditions, in the same order as the scheme in (C). Error bars represent the standard deviation from at least 3 independent replicates.

In this report, we generated D81A, D81F, and D81K EF-Tu mutants, to investigate the consequences of perturbing a conserved switch II Mg^2+^-coordinating residue. The GTP binding pocket in EF-Tu contains 5 conserved motifs: the P-loop, switches I and II, and the G4 and G5 motifs, which together mediate nucleotide phosphate binding, γ-phosphate and Mg^2+^ sensing, and guanine recognition (Fig. 1A) [23]. In full-length *E. coli* EF-Tu numbering including methionine, these motifs correspond approximately to residues 19 to 26, 41 to 63, 81 to 101, 136 to 139, and 174 to 177, respectively [17]. The D81 lies within switch II and coordinates a Mg^2+^ ion near the γ-phosphate (Fig. 1B) [24]. Although D81 is strongly conserved and positioned at the center of the G domain, it remains underexplored because mutations at this site can impair EF-Tu stability [25]. Its importance is further supported by disease-associated mutation of the corresponding residue in human eEF1A2 and by the emergence of switch I/switch II regions as druggable sites in other GTPase families, including K-RAS [26,27]. Here, we used a 2-step AlphaFold2 clustering strategy to ask whether D81 perturbation changes the predicted conformational and sequence landscape. Structures were first sorted by the global interdomain orientation of helix 183 to 199, then by local variation in switch I loop 54 to 58, separating global and local transitions that would otherwise be conflated and linking each conformational cluster to its contributing sequences. This design allowed us to ask 2 questions. First, whether perturbing a conserved residue changes only the predicted structures, or also the sequence neighborhoods and organismal diversity. Second, whether the local GTP-pocket state and the global domain conformation are tightly or loosely coupled across the predicted ensemble, since the relationship between these 2 conformational coordinates bears directly on how decoding is transmitted to hydrolysis. Together, these questions test whether invariant positions, far from being uninformative, may be revealing probes of hidden sequence and conformational diversity.

## Results

### Mutagenesis and protein synthesis

D81 mutations were introduced into the EF-Tu pET-21b(+) plasmid using the Q5 Site-Directed Mutagenesis Kit (New England Biolabs) with primers from Integrated DNA Technologies. Under standard His-tag affinity purification protocol using a Cytiva HisTrap Fast Flow (FF) column and an imidazole gradient (detail in supplemental information), the D81A mutant showed extensive degradation (Fig. S2A), in agreement with literature report [25]. Adding 5 μM GDP to lysis and chromatography buffers stabilized the protein but substantially reduced yields of all D81 variants, likely due to increased inclusion body formation (Fig. S2B) [28]. To improve recovery, osmolytes (1 M sorbitol and 25 mM betaine) were included during cell culture, which enhanced overall yields (Fig. S2C). For D81A and D81K, an additional purification step using a Cytiva HiTrap DEAE Sepharose Fast Flow (FF) anion-exchange column with a 0 to 500 mM NaCl gradient was required, while wild type (WT) and D81F reached sufficient purity without it (Fig. S2D).

### GTPase assays of D81 mutants

GTP hydrolysis was quantified using N-methylanthraniloyl (mant)-labeled GTP and fluorescence thin-layer chromatography (TLC). The mant-GDP percentage was calculated as GDP spot intensity divided by the sum of GDP and GTP spot intensities [mant-GDP (%) = GDP / (GDP + GTP) × 100], with higher mant-GDP percentage indicating increased hydrolysis [29]. Six conditions were tested (Fig. 1C and D): EF-Tu alone or with tRNA, with or without ribosome, and with mRNA paired with cognate or near-cognate tRNA. Without ribosome, hydrolysis was minimal, consistent with EF-Tu’s low intrinsic GTPase activity. Ribosome addition substantially increased hydrolysis, confirming its role as a GTPase-activating factor. Aminoacyl-tRNA slightly suppressed hydrolysis in the absence of mRNA, while cognate ternary complex enhanced GTPase activity and near-cognate tRNA suppressed it, demonstrating coupling between GTP hydrolysis and proofreading. All original TLC images are included in Figs. S3 to S6. D81A, D81K, and D81F exhibited hydrolysis patterns comparable to WT, indicating that D81 does not substantially affect the core GTPase mechanism during cognate versus near-cognate tRNA discrimination. This indicates that D81 substitution does not abolish hydrolysis under these conditions, although endpoint assays cannot exclude kinetic effects at individual steps (see Discussion).

### AlphaFold modeling and 2-step structure clustering

We modeled all EF-Tu·GTP and EF-Tu·GDP complexes with AlphaFold3 to gain mechanistic insight of the experimental observations. All predictions generated high-confidence structures (Figs. S7 to S10), reproducing experimentally observed open and close conformations involving the position of domain I relative to domains II to III, indicating that D81 substitutions do not lock the conformational flexibility required for GTP hydrolysis. However, Mg^2+^ coordination with residue 81 was consistently weaker and more variable in mutants, with D81K•GDP showing the most disrupted binding (Tables S1 and S2). In addition, when a sarcin–ricin loop (SRL) was added in modeling, its position was stable between GTP and GDP forms in WT but shifted specifically in the GTP-bound mutants while GDP-bound forms retained WT SRL positioning. However, AlphaFold predictions carry inherent limitations: They reflect MSA composition rather than direct sampling of a free-energy landscape. The modeling also lacks the full ribosomal context and solvent environment, so these structural inferences remain to be confirmed by experiments.

**Fig. 2.**
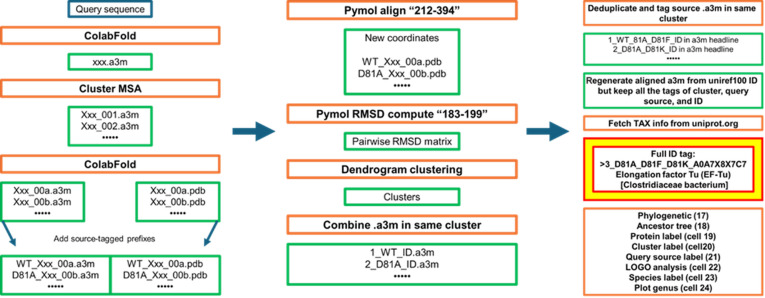
Workflow of structure-guided sequence, phylogenetic, and ecological analysis. Step 1: Generate high-confidence predicted structures and tag each by query source. Step 2: Apply 2-step clustering to all structures, tagging both.a3m and .pdb files with conformational cluster IDs. Step 3: Combine all sequences with deduplication and annotate with protein identity and taxonomic information, producing the final tagged dataset for downstream analysis. Blue boxes indicate query sequences, orange boxes indicate transformation modules, and green boxes indicate input and output data. Numbers in parentheses in the final box correspond to cell numbers in the Jupyter notebook deposited at github.com/ywang6000/EF-Tu-structure-clustering.

**Fig. 3.**
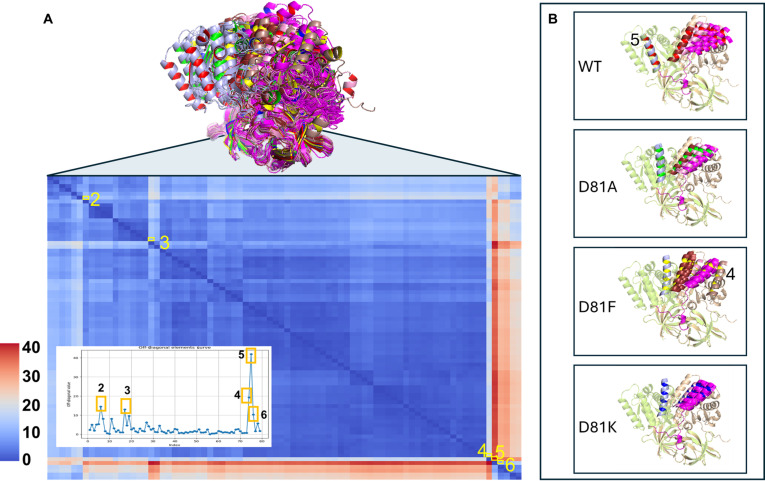
Two-step clustering identifies 6 distinct conformational states of domain I relative to domains II to III. (A) Dendrogram of pairwise root mean square deviation (RMSD) values between all predicted structures. The inset shows values immediately above the diagonal of the RMSD matrix, where each point exceeding the threshold of 10 Å marks a conformational cluster boundary. Transition points are indicated in both the RMSD heatmap and the inset. (B) High-confidence predicted structures from wild-type and D81A/F/K queries. Solid ribbons represent helix 183 to 199, which connects domain I to domain II. Outer ribbon colors indicate conformational cluster (clusters 1 to 6: brown, pink, magenta, golden, steel blue, and light blue); inner colors indicate query source (wild-type [WT]: red; D81A: green; D81F: yellow; D81K: dark blue). The 2 background EF-Tu structures are pdb 1EFT and 1EFC for close and open forms, respectively.

To dissect how D81 substitutions reshape the conformational and evolutionary landscape of EF-Tu, we developed a structure-guided sequence analysis pipeline that integrates AlphaFold2 predictions, structural clustering, and phylogenetic annotation into a single unified framework (Fig. 2) [3,7,30]. We first generated MSA-seeded AlphaFold2 predictions using 4 queries: WT, D81A, D81F, and D81K, then retained only high-confidence predictions (pLDDT > 70). All predicted structures and their sequence files were pooled into a common repository with source-tagged identifiers (e.g., wt_xxx.a3m, wt_xxx.pdb, D81A_yyy.a3m, D81A_yyy.pdb, etc.). Conformational clustering was then performed in 2 steps: Structures were first globally aligned over the rigid scaffold of domains II to III (residues 212 to 394) and then clustered by pairwise RMSD of the domain I terminal helix (residues 183 to 199), which is the precise structural element that reports interdomain displacement. Clusters were defined by a threshold of 10 Å on the sequential off-diagonal RMSD trace, yielding 6 distinct conformational states (Fig. 3A). Although all predicted structures sampled both open and closed conformations of helix 183 to 199, the WT query produced the most open state (cluster 5), which aligned well with the x-ray structure 1EFC (RMSD = 3.525 Å) [31]. In contrast, the mutant queries showed more restricted interdomain orientations (Fig. 3B and Fig. S11): Most populated a smaller-angled open state (cluster 6), whereas D81F adopted a distinct closed orientation at the opposite extreme (cluster 4). Clusters 2 and 3 were sampled by all 4 queries, and cluster 1 by 3 queries but not D81K. Cluster 3 aligned well with the x-ray structure 1EFT (RMSD = 3.382 Å) [24], providing partial validation of the predicted conformational states, although experimental confirmation are required for the intermediate states. Notably, these conformational states became apparent only after clustering; without it, predictions from all 4 queries were dominated by the closed form.

After clustering, all of the sequences are pooled together deduplicated with tagging of conformation and consolidated query source. For example, “>1_WT_D81A_D81K_xxx” means the uniref ID xxx is pooled into a3m files from 3 query seeds (WT, D81A, and D81K) and predicted structure of cluster 1. Only one of the IDs is used to construct the final aligned sequence, and proteins and taxonomic origins are fetched from unipro.org. The key milestone is the full sequence label, which encodes conformation cluster, query source, and UniRef100 ID in a single header; for example, >3_D81A_D81F_D81K_A0A7X8X7C7 Elongation factor Tu [Clostridiaceae bacterium] shows that 3 is the conformational cluster, D81A_D81F_D81K indicates which queries recruited this sequence, A0A7X8X7C7 is the UniProt ID, and Elongation factor Tu [Clostridiaceae bacterium] identifies the protein and its taxonomic origin. This unified label drives all downstream phylogenetic, species, and ecological analyses by simple keyword filtering (Fig. 2).

To resolve finer conformational variation within the 6 global clusters, we sorted structures by the open and closed states of loop 54 to 58 in switch I, which gates the GTP binding pocket (Fig. S12). Across most clusters, GTP binding pocket conformation showed no consistent correlation with global interdomain state: Structures within the same cluster populated both open and closed pocket forms, indicating that global and local conformational transitions are largely independent. This independence was most apparent in cluster 3, where open and closed pocket forms were both represented abundantly. Coupling emerged only in clusters 5 and 6, which correspond to the most D-open forms and showed a tendency toward the switch-open state, indicating that local pocket opening and global domain opening become correlated mainly at the open, tRNA-release extreme. These results justify the 2-step clustering strategy: Global clustering alone would obscure this local conformational diversity by expanding RMSD range, and switch I state cannot be inferred from interdomain orientation alone. Focused short-range RMSD on functionally relevant segments therefore provides finer resolution than long-range alignment for capturing local conformational heterogeneity.

### Structure-guided phylogenetic and ecological analysis

**Fig. 4.**
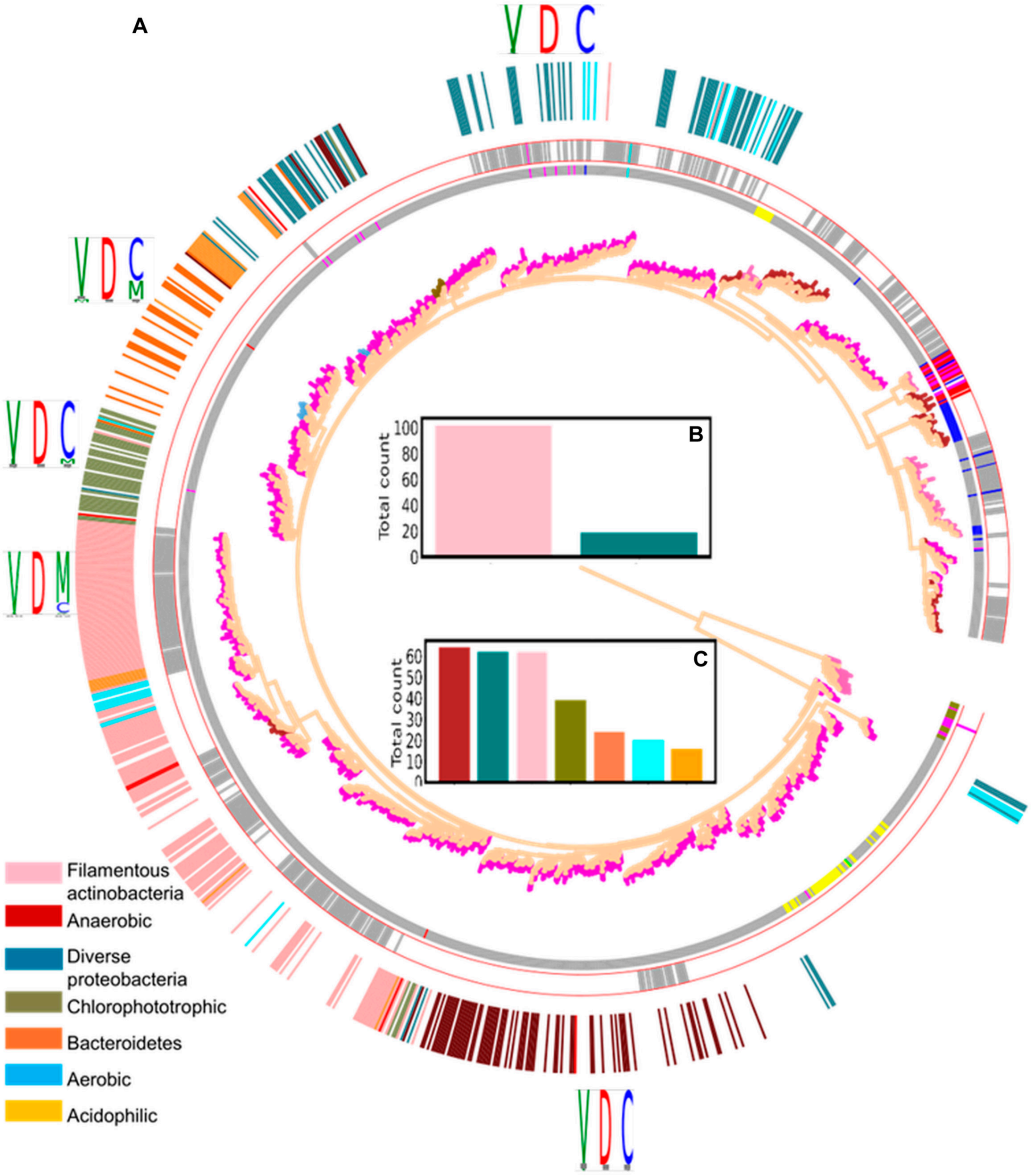
Phylogenetic and ecological analysis of the final deduplicated, tagged multiple sequence alignment (MSA) collection. (A) Phylogenetic tree with midpoint rooting. The innermost thin and thick rings indicate protein identities for D81K-sourced and wild-type (WT)-sourced sequences, respectively. Leaf tip colors indicate conformational cluster assignment, following the same color scheme as Fig. 3. The outer ring with long stems annotates the most populated species across the full tree. Sequence logos placed by the species notes are flanking position 81 and indicate conservation in the surrounding region. (B) Significant species analysis for sequences recruited by both WT and D81K queries. (C) Significant species analysis for sequences recruited by D81K but not WT.

The final structure-filtered, deduplicated, labeled MSA generated a maximum likelihood phylogenetic tree (Fig. 4A) [32]. The tree resolved into 2 major branches (middle point root): one representing mostly EF-Tu (1,197 leaves), predominantly annotated with magenta leaves (experimental close form), and the other representing mostly EF-1α (21 leaves, medium close form), enriched in pink leaves. Minor clusters represented additional conformational states in light blue, golden, and brown leaves. Protein identities were annotated with 2 inner rings: a thin inner ring indicating D81K decoding proteins, and a wider ring indicating WT-decoding proteins. Sequences from D81A and D81F largely overlap to D81K and therefore are not discussed separately. Most sequences decode canonical EF-Tu (gray), with smaller subsets classified as mitochondrial EF-Tu (blue), chloroplastic EF-Tu (yellow), EF-1α (dark green), unknown (red), and general translational G protein (magenta).

**Fig. 5.**
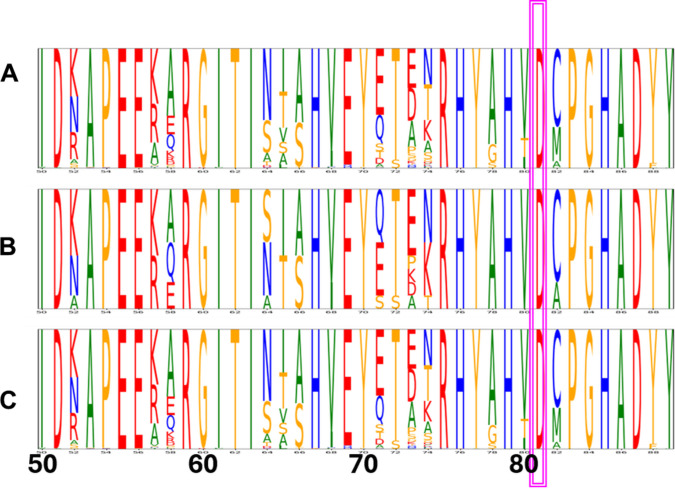
D81 remains invariant across all multiple sequence alignment (MSA) collections despite mutant query. (A) A total of 379 sequences common to wild-type (WT) and D81K queries; (B) 9 sequences unique to WT query; (C) 717 sequences unique to D81K. Position 81 is highlighted with a frame.

Mutant queries recruited substantially more sequences than WT: 902 vs. 416 out of 1,318 total. Among EF-Tu entries, 379 sequences were shared between WT and D81K, 717 were unique to D81K, and only 9 were unique to WT. Shared sequences were predominantly from filamentous species, while sequences unique to D81K spanned a broader ecological range including anaerobic, chlorophototrophic, acidophilic, and thermophilic organisms (Fig. 4B and C). Strikingly, despite this expanded ecological and phylogenetic diversity, residue 81 remains aspartate across virtually all retrieved sequences in every MSA collection for EF-Tu proteins (Fig. 5). The broader ecological range recovered by mutant queries reflects a shift in MSA recruitment, not direct evidence that those organisms naturally tolerate D81 substitution, since position 81 remained aspartate across the retrieved MSAs.

## Discussion

The AlphaFold3 models suggest a possible structural basis for the preserved GTPase activity. Because SRL positions EF-Tu’s catalytic His85 (equivalent to His84 in literature without the first methionine residue) through its A2662 contact for ribosome-induced GTPase activity [14–16], the GTP-specific SRL displacement predicted in the mutants may indicate a kinetic or downstream effect rather than a block in catalysis itself. This would be consistent with the endpoint GTPase assay in Fig. 1, which detects total turnover but not the rate of individual steps. The catalytic geometry itself appears preserved: As shown in Fig. S13, His85 is positioned similarly in WT and mutants in both the GTP and GDP states. This also reconciles the weakened Mg^2+^ coordination with preserved hydrolysis, because the Mg^2+^ perturbation is most pronounced in the GDP state, whereas His85 has already rotated away from the nucleotide as in WT; the defect therefore falls in a postcatalytic state rather than at the transition state. In this view, preserved hydrolysis does not require compensatory rearrangements elsewhere in the active site; rather, the catalytically relevant GTP-state geometry is itself largely unperturbed. We could not assess the position of the catalytic water, which AlphaFold3 does not model explicitly, so we cannot exclude subtle changes in the water network or transition-state geometry that an endpoint assay would not detect. The TLC assay also measures ribosome-stimulated hydrolysis only and does not assess nucleotide exchange, aminoacyl-tRNA binding, or ribosome dissociation. Because D81 coordinates the Mg^2+^ that governs nucleotide affinity, an effect on GDP/GTP exchange remains plausible and will require direct kinetic and exchange measurements. Recent comparisons of AlphaFold3 and template-based models have similarly shown that accurate overall folds do not always capture functional mutation effects, which need experimental constraints [33].

Meanwhile, AlphaFold2 with clustering showed narrower conformational range sampled by mutant queries (Fig. 3B), suggesting that D81 may contribute to EF-Tu’s conformational accessibility. One possibility is that the conserved aspartate acts not only as a catalytic anchor but also as an element permitting the full range of interdomain flexibility used during tRNA accommodation. Because these inferences rest on AlphaFold sampling rather than direct measurement, it remains a hypothesis to be tested experimentally. AF-Cluster-based conformational sampling can predict states that are not necessarily evolutionarily selected [34], and machine learning analyses on limited biological data require domain knowledge and careful validation [35]. In our case, however, the perturbation is functionally guided rather than random: D81 is a conserved switch II residue involved in Mg^2+^ coordination, and all 3 mutants retained GTPase activity under the tested conditions. Our central conclusion is therefore not that AlphaFold2 alone proves the existence of an evolutionarily relevant conformational state but that D81 perturbation exposes otherwise hidden links among conformational sampling, MSA recruitment, and organismal diversity that are not apparent from WT searches.

Phylogenetic and ecological analyses point to a third possible role of D81 beyond Mg^2+^ coordination and conformational restriction: D81 may mark a broader evolutionary constraint. Mutant queries preferentially recruited sequences from ecologically distinct environments, suggesting that perturbing this conserved site changes the region of natural sequence space recovered by MSA search, although the mechanistic basis of this effect remains unresolved. Together, these observations reveal a paradox. D81K, D81A, and D81F were not present in the retrieved natural sequences, yet introducing these substitutions in the query reshaped MSA recruitment and conformational sampling. One possible interpretation is that mutating a deeply conserved residue perturbs the coevolutionary signal used during MMseqs2 retrieval, exposing sequence diversity that is less apparent from the WT query alone [36–38]. Related computational–experimental and simulation-based studies have shown that single-residue substitutions can strongly alter protein behavior by changing conformational plasticity, catalytic activity, stability, or specific binding interactions [39–42]. Together with our results, this supports the idea that conserved residues such as D81 can serve as probes of hidden sequence, ecological, conformational, and functional constraints.

This principle may extend across the GTPase superfamily. D81 is the conserved aspartate of the switch II DXXG motif and is near-invariant not only in translational GTPases but also in Ras, Rho, and the larger G-protein superfamily [23]. Similar studies of Ras-family GTPases show that switch II dynamics can reshape GTP/GDP-bound conformational ensembles [43]. This framework could also identify other D81-like residues: conserved sites that tolerate substitution yet reshape MSA recruitment and conformational sampling when perturbed. Testing such residues will be an important direction for future work. More broadly, conserved residues that anchor coevolutionary networks may reveal hidden sequence or conformational diversity, of potential value for emerging drug-development efforts targeting switch I/II and their dynamics in Ras and Rho proteins [26,44].

## Conclusion

This study makes 3 main contributions. First, we produced and biochemically characterized the EF-Tu mutants D81A, D81F, and D81K, previously inaccessible due to instability, and showed that all 3 retain GTPase activity, suggesting that D81 is biochemically tolerant of substitution. Second, we developed a 2-step structure-guided clustering strategy that separates global interdomain motion from local GTP-pocket rearrangement, and links each predicted conformation to its contributing sequences and their taxonomic and ecological context. We found that the 2 conformational coordinates are largely uncoupled, becoming more correlated only at the fully open, tRNA-release extreme (Fig. S12). Third, using this approach, we found that mutant queries recruit broader sequence and ecological diversity than WT, yet position 81 remains aspartate across virtually all retrieved sequences regardless of query. While existing approaches focus on conformational diversity [6–8,10,30,34], we expanded these approaches by sorting complex structural ensembles and linking them to phylogenetic and ecological context.

## Materials and Methods

Additional materials and methods are provided in the Supplementary Materials.

### AlphaFold3 modeling

Modeling was performed using the AlphaFold3 server (https://alphafoldserver.com/). Inputs comprised the following:

*Protein:* *E. coli* EF-Tu (tufB; UniProt P0CE48), modeled as WT and as the D81A, D81F, and D81K point substitutions.

*RNA (SRL)*: 5′-CUGCUCCUAGUACGAGAGGACCGGAGUGGA-3′

RNA (22 nt): 5′-ACAGAGGGAACCGGCGGAAAGG-3′. This RNA was included to help the SRL dock at its structurally confirmed position, likely by occupying an EF-Tu surface that, in the context of the full ribosome, is bound by ribosomal RNA.

*Ligands/ions:* 1 Mg^2+^ ion and 1 molecule of GTP (or GDP).

### AlphaFold2 modeling

Modeling was through ColabFold and AF-Cluster (full notebook deposited at github.com/ywang6000 with detailed readme for reproducing the results).

*MSA generation and AlphaFold2 prediction.* MSAs were generated using ColabFold (version 1.5.5) with the WT EF-Tu sequence (UniProt ID P0CE48) and 3 D81 mutant sequences (D81A, D81F, and D81K) as independent queries. For each query, ColabFold queried the MMseqs2 server against UniRef and environmental databases with default parameters, producing query-specific.a3m alignment files. No preassembled MSA was used; each query independently generated its own MSA through the ColabFold pipeline.

*MSA clustering and structure prediction.* Each query-specific.a3m alignment was clustered using DBSCAN with 3 epsilon ranges [min_eps = 3,10.5, 18, max_eps = 18, 25.5, 33.0 step = 1.5] to find the best epsilon range on 25% data, then find the best epsilon value in that range in the full dataset to generate the maximum cluster numbers, such as eps = 12, 409 clusters from 12,585 sequence. This is modified from the original python from Wayment-Steele et al. [7] Clusters containing more than 7 sequences (>4-kb total sequence content) were retained for structural prediction. ColabFold (version 1.5.5) was then run on each retained cluster, producing predicted structures with associated pLDDT confidence scores. Only structures with mean pLDDT > 70 were retained for downstream analysis.

*Source tagging and pooling.* All retained .a3m and.pdb files were pooled into a single repository with source-tagged identifiers indicating the query of origin (e.g., wt_xxx.a3m, D81A_yyy.pdb).

*Two-step conformational clustering.* Pooled structures were clustered in PyMOL using a hierarchical 2-step approach. First, structures were globally aligned to a reference using residues 212 to 394 (domains II to III) to remove rigid-body orientation variation. Second, pairwise RMSD was calculated over residues 183 to 199 (the domain I terminal helix connecting domain I to domain II) to quantify interdomain displacement. Clusters were defined using a 10-Å threshold on the sequential off-diagonal RMSD trace, yielding 6 global conformational clusters. Within each global cluster, a second clustering step was applied over residues 54 to 58 (switch I loop) to resolve local GTP binding pocket conformations.

*Sequence pooling and annotation.* Sequences contributing to retained structures were extracted from their.a3m files and pooled across all clusters. Sequences were deduplicated by UniRef100 ID, with query sources consolidated into a single tag (e.g., WT_D81A_D81K_xxx). Protein identity and taxonomic origin were retrieved from UniProt.org via API queries using the UniRef100 ID, producing the final unified sequence label (e.g., >3_D81A_D81F_D81K_A0A7X8X7C7 Elongation factor Tu [Clostridiaceae bacterium]).

*Phylogenetic and ancestral sequence analysis.* Maximum likelihood phylogenetic trees were constructed from the final deduplicated, labeled MSA using IQ-TREE 2 [32]. The best-fit substitution model was selected automatically using ModelFinder (-m TEST), branch support was assessed with 1,000 ultrafast bootstrap replicates (-bb 1000), and the number of threads was determined automatically (-nt AUTO). Ancestral sequences were then reconstructed on the resulting tree topology using the empirical Bayesian method implemented in IQ-TREE 2 (--ancestral). Trees were visualized and annotated using iTOL [45].
