# Supplementary material for: D81 Mutants Reveal Hidden EF-Tu Diversity while Natural Sequences Preserve Aspartate
Source: Comput Struct Biotechnol J. 2026 Jul 10;35(1):0167. doi: 10.34133/csbj.0167 (PMC13351119; doi:10.34133/csbj.0167)
Supplement: Supplementary 1 — Methods Figs. S1 to S13 Tables S1 and S2 [file csbj.0167.f1.pdf]

Supporting Information

D81 Mutants Reveal Hidden EF-Tu Diversity while Natural Sequences Preserve Aspartate

Jordan L. Johnson,[2] Yuhong Wang[1*]

[1]*Department of Chemistry, University of Houston, Houston, TX 77204, USA*

[2]*Department of Biology and Biochemistry, University of Houston, Houston, TX 77204, USA*

**Corresponding authors. Email: ywang60@uh.edu (Y.W.),*

TABLE OF CONTENTS

1. Methods.
2. AlphaFold3 predicted EF-Tu complexes bound to GTP or GDP with Mg^{2+}, with Figure S1.
3. PAGE analysis of EF-Tu WT and mutant preparations, with Figure S2.
4. Original TLC images for Four EF-Tu species in Figure 1, with Figures S2-S6.
5. AlphaFold3 reproduces the experimentally observed closed and open conformations of WT, D81A, F, K mutated EF-Tu, correlating with GTP- and GDP-bound states, with Figure S7-S10.
6. Conformational variation of domain I tracked by its terminal helix (residues 183–199), shown for WT (A), D81A (B), D81F (C), and D81K (D), with Figure S11.
7. Conformations of the GTP binding pocket show no consistent correlation with global conformational clusters, with Figure S12.
8. AlphaFold3-predicted His 84 position in EF-Tu complexes bound to GTP or GDP, with Figure S13.
9. Folding confidence metrices for EF-Tu (chain 0) with nucleotide (chain 1) and Mg^{2+} (chain 2), with Table S1.
10. Folding confidence metrices for EF-Tu (chain 0) with nucleotide (chain 1), Mg^{2+} (chain 2), 22-nt RNA (chain 3), and 30-nt SRL (chain 4). With Table S2.

METHODS

EF-Tu D81 mutants expression and purification

The D81 mutants were introduced into wild type EF-Tu pET-21b(+) plasmid using the Q5® Site Directed Mutagenesis Kit from New England Biolabs and mutagenesis primers ordered from Integrated DNA Technology. Cells from an overnight culture were incubated in LB containing 1 M sorbitol and 25 mM betaine until mid-log phase. Mid-log-phase cells (OD600 ~0.6–0.8) were induced with 0.1 mM IPTG and harvested after 18 hours of growth at 30 °C. The cell pellets were collected by centrifugation at 4,000 ×*g* for 30 minutes at 4 °C. The pellets were resuspended in lysis buffer (50 mM Tris-HCl, 10 mM $MgCl_2$, 5 mM BME, 5 µM GDP, 15 % glycerol, 60 mM NH_4Cl, pH 7.6) containing a small amount of lysozyme crystals and incubated at -20 °C overnight. The cells were thawed then lysed using sonication at 20% power amplitude (e.g., 10 seconds on, 20 seconds off) for 2 minutes. The lysate was clarified by centrifugation at 15,000 ×*g* for 30 minutes at 4°C. The cleared lysate was loaded onto a HisTrap Fast Flow (FF) 5 mL column (Cytiva) pre-equilibrated with binding buffer (50 mM Tris-HCl, 10 mM $MgCl_2$, 5 mM BME, 5 µM GDP, 300 mM NaCl, pH 7.6) and allowed to cycle for 1 hour. The column was washed with 10 column volumes of wash buffer (50 mM Tris-HCl, 10 mM $MgCl_2$, 5 mM BME, 5 µM GDP, 300 mM NaCl, pH 7.6) to remove nonspecific proteins. The target proteins were eluted at approximately 250 mM imidazole using a 5–500 mM gradient on a GE ÄKTA Explorer 10 FPLC system. Eluted fractions were analyzed by SDS-PAGE to confirm purity. Wild type and D81F were subsequently buffer-exchanged into storage buffer (50 mM Tris-C, 10 mM $MgCl_2$, 5 mM BME, 5 µM GDP, 100 mM NaCl, pH 7.6) while D81A and D81K underwent an additional purifying step by diluting the sample with Milli-Q water before loading on a HiTrap DEAE Sepharose FF column 1 mL (Cytiva) and cycling overnight. The column was washed with 10 column volumes of wash buffer (50 mM Tris-HCl, 10 mM $MgCl_2$, 5 mM BME, 5 µM GDP, pH 7.6). The target proteins were eluted at approximately 300 mM NaCl using a 0–500 mM gradient on a GE ÄKTA Explorer 10 FPLC system.

Ribosome and translational factors preparation

70S ribosomes and the ribosome factors were purified from *E. coli* MRE600 following previously described protocols (Altuntop, M.E., Ly, C.T., and Wang, Y. *Biophys. J.* **99**, 3002-3009, 2010). The cleared supernatant was layered onto a pre-cooled 1.1 M sucrose cushion in Buffer II (20 mM Tris-HCl, pH 7.6; 10 mM $MgCl_2$; 100 mM NH_4Cl; 6 mM β-mercaptoethanol (BME); 0.5 mM EDTA) at a 1:1 volume ratio. Ribosomes were pelleted by ultracentrifugation using a Beckman XL-80 ultracentrifuge with a Type 45 Ti Fixed-Angle rotor at 120,000 ×*g*, 4 °C, for 20 hours. The resulting

pellet was gently rinsed with Buffer II and resuspended in the same buffer. The sucrose cushion ultracentrifugation step was repeated twice more, after which the ribosomes were resuspended in a minimal volume of Buffer II. The concentration of ribosomes was determined by measuring UV absorbance at 260 nm, using the conversion factor of 1 A260 unit = 23 pmol of 70S ribosomes. The purified ribosome solution was aliquoted, flash-frozen in liquid nitrogen, and stored at -80 °C.

fMet-tRNA^{fMet} preparation

E. coli tRNA^{fMet} was overexpressed in BL21 Star (DE3)pLysS cells from a pBluescript II SK (+) plasmid (Genscript) and purified on a Sepharose 4B column with a reverse ammonium sulfate gradient (methionine-accepting activity: 500 pmol/A260 unit). Recombinant 6×His-tagged *E. coli* methionyl-tRNA synthetase and methionyl-tRNA^{fMet} formyltransferase were expressed and purified (Altuntop, M.E., Ly, C.T., and Wang, Y. *Biophys. J.* **99**, 3002-3009, 2010). The formyl donor, 10-formyltetrahydrofolate, was prepared as described before (Dubnoff, J. S. and Maitra, U. *Proc. Natl. Acad. Sci. USA* **68**, 318-323, 1971). Aminoacylation and formylation were performed as a one-pot reaction containing 100 mM Tris-HCl (pH 7.5), 4 mM ATP, 20 mM $MgCl_2$, 10 mM KCl, 150 μM L-methionine, 750 μM neutralized formyl donor, 7 mM BME, 20 μM tRNA^{fMet}, 12 μM methionyl-tRNA synthetase, and 16 μM formyltransferase. The mixture was incubated at 37 °C for 15 min, acidified to 0.3 M sodium acetate (pH 5.0), and purified by phenol-chloroform extraction, gel filtration (NAP-10, Cytiva), and ethanol precipitation. The final fMet-tRNA^{fMet} was dissolved in 2 mM sodium acetate (pH 5.0) and stored at -80 °C. The charging efficiency was estimated at 30–50% using radiolabeled methionine.

Preparation of Ribosome Initiation Complex (ribosome IC)

The ribosome initiation complex is prepared by mixing 1 μM ribosomes, 1.5 μM each of initiation factors (IF1, IF2, IF3), 2 μM mRNA (decode “MF” for the first two amino acids), 4 μM charged fMet-tRNA^{fMet}, and 4 mM GTP, then it was preincubated at 37 °C for 15 minutes.

GTP hydrolysis assay with thin layer chromatography (TLC)

Reaction mixtures contained 1 μM EF-Tu (wild type or mutant), 400 μM mant-GTP (Sigma-Aldrich), 1× TAM10 buffer (20 mM Tris-Cl pH 7.5, 10 mM MgAc, 30 mM NH_4Cl, 70 mM KCl, 5 mM EDTA, 7 mM BME), 0.5 μM of ribosomes IC (cognate, near-cognate, or no mRNA) or no ribosome IC, 0.5 μM charged Phe-tRNA^{Phe} or no tRNA. While in dim lighting, the reaction tubes were incubated at 37 °C for 10 minutes. After incubation, 0.8 μL of each reaction mixture was carefully spotted onto a TLC plate using a micropipette. The plates were air-dried for approximately 10

minutes before being placed into TLC chambers. The mobile phase consisted of 3 mL 100% ethanol, 2 mL aqueous ammonia (saturated, ~29%), 0.5 mL 1 M ammonium acetate, 4.5 mL H_2O, and 30 mg EDTA in H^+-form. TLC plates were incubated in the chambers until the mobile phase traveled the full length of the plate (~60 minutes). After development, the plates were removed, air-dried, and placed in a dark chamber for visualization using a UVP UVGL-25 Compact 4-watt UV Lamp with a spectral range of 254–365 nm. Images of the TLC plates were captured and analyzed using ImageJ. Regions of interest (ROIs) corresponding to separated spots for mant-GTP and its hydrolysis product of mant-GDP were defined. Fluorescence intensities were measured, and the percentage of hydrolyzed mant-GTP was calculated as the ratio of the intensity of mant-GDP to the sum of the intensities of mant-GDP and mant-GTP.

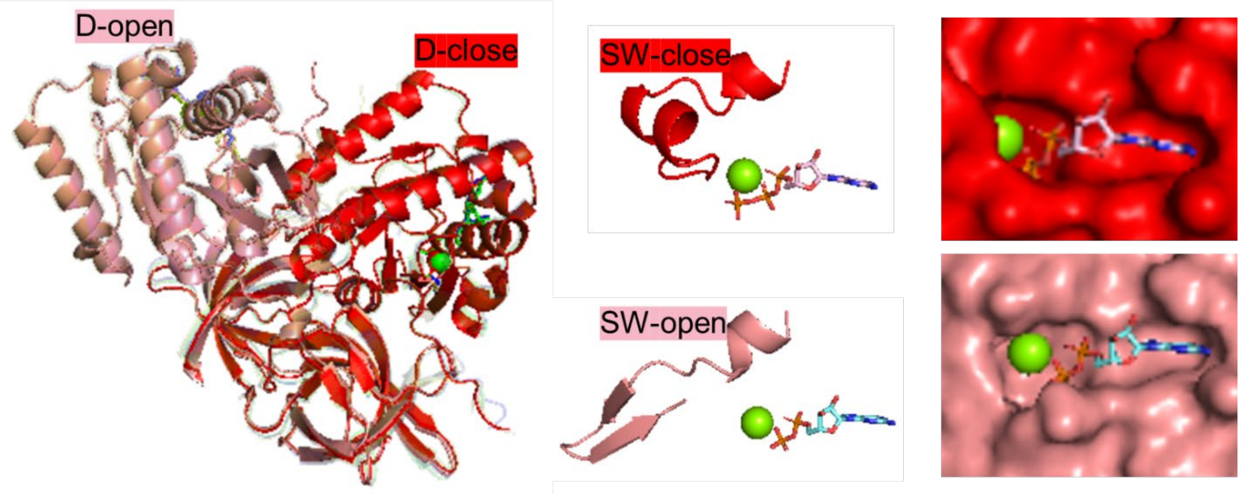

Figure S1. AlphaFold3-predicted EF-Tu complexes bound to GTP or GDP with Mg^{2+}. The D and SW regions mark two conformationally flexible, allosterically connected sites. D-open and D-close describe the global orientation of domain I relative to domains II–III, while the SW region is a flexible loop flanking the GTP binding pocket that reorients to release Pi following GTP hydrolysis. A 30-nucleotide sarcin–ricin loop (SRL) and a 22-nucleotide RNA were included in the modeling but are not shown for clarity. The D-close/open predicted structures agree with pdb ID 1eft (RMSD 3.382) and 1efc (RMSD 3.525), respectively.

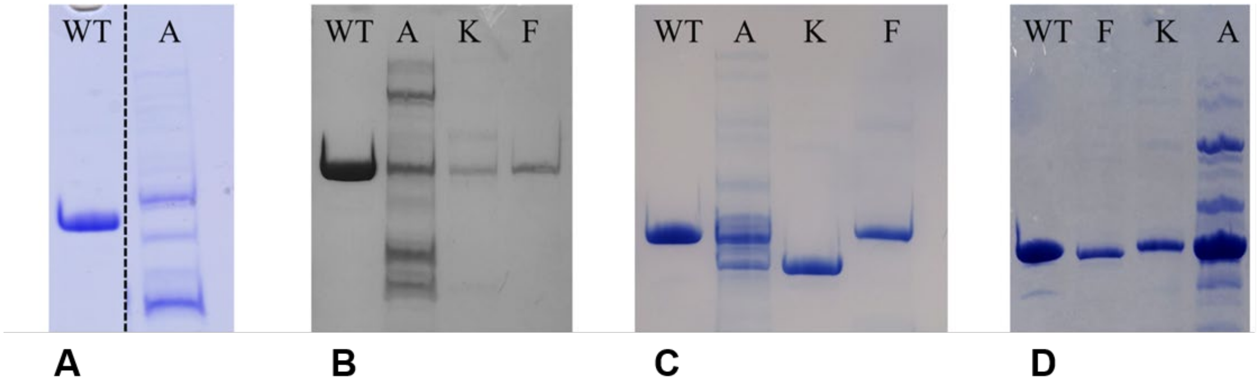

Figure S2. PAGE analysis of EF-Tu WT and mutant preparations. (A) Standard protocol led to D81A degradation. (B) Addition of 5 μM GDP to lysis and purification buffers stabilized D81A but reduced yields for all mutants. (C) Supplementing culture media with 1 M sorbitol and 25 mM betaine improved recovery. (D) An additional ion-exchange purification using a 0–500 mM NaCl gradient was required to purify D81A and D81K, whereas WT and D81F yielded adequately without this step.

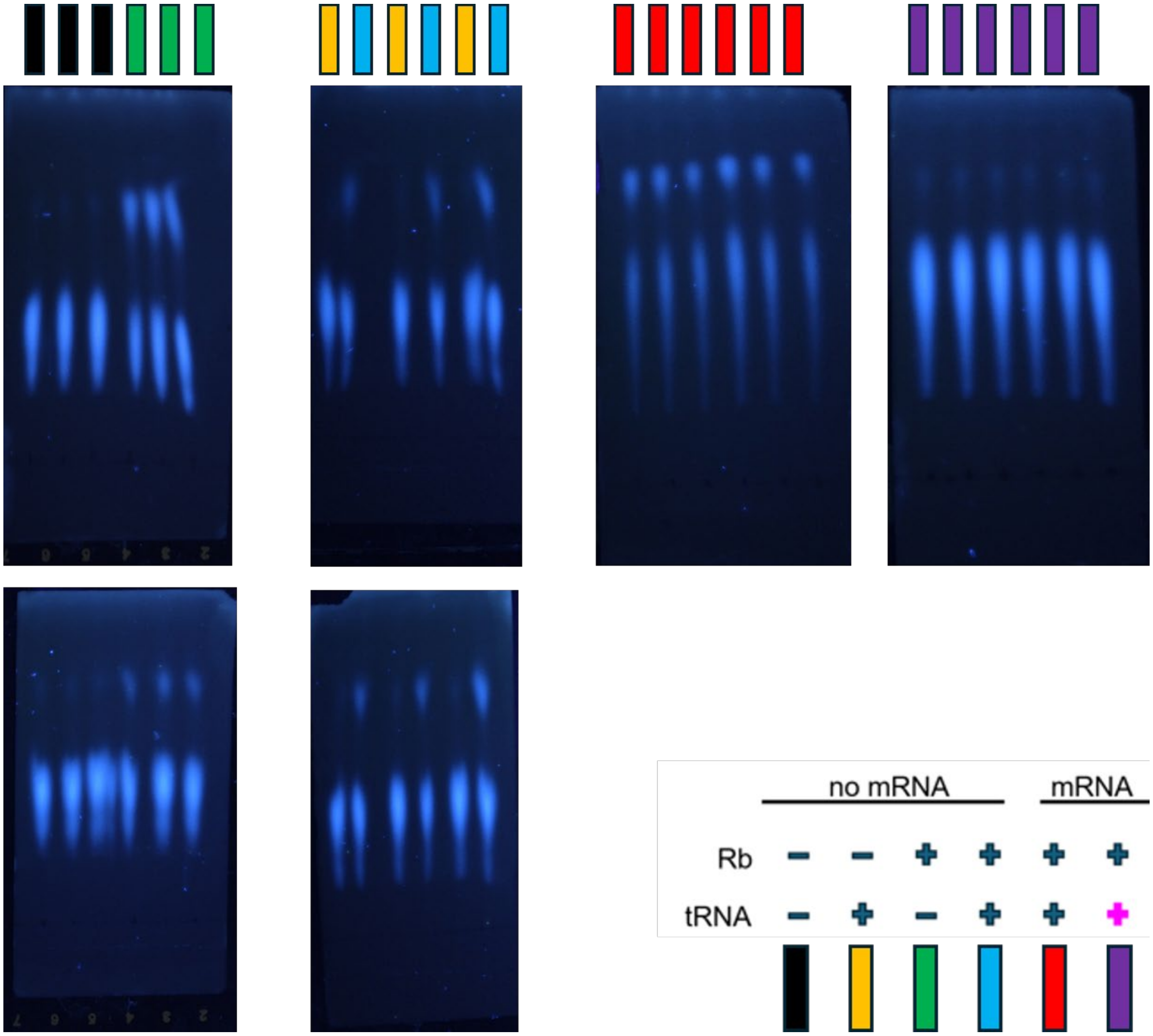

Figure S3. Original TLC images of WT EF-Tu for Figure 1 in the main text. The experimental conditions are indicated by the colored bars in each column (bottom-right panel). If more than one triplicate is shown, they are displayed beneath the first set.

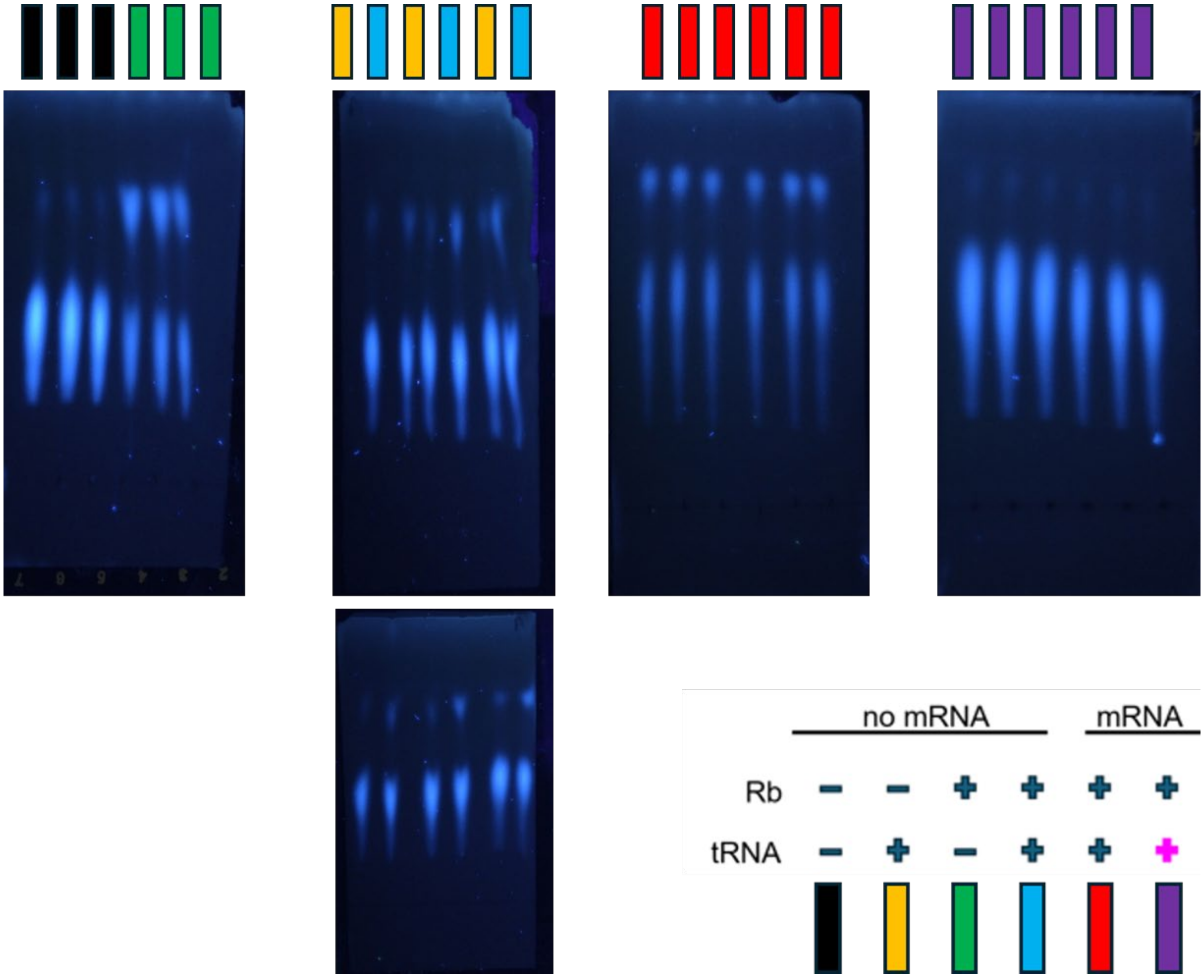

Figure S4. Original TLC images of D81A EF-Tu for Figure 1 in the main text. The experimental conditions are indicated by the colored bars in each column (bottom-right panel). If more than one triplicate is shown, they are displayed beneath the first set.

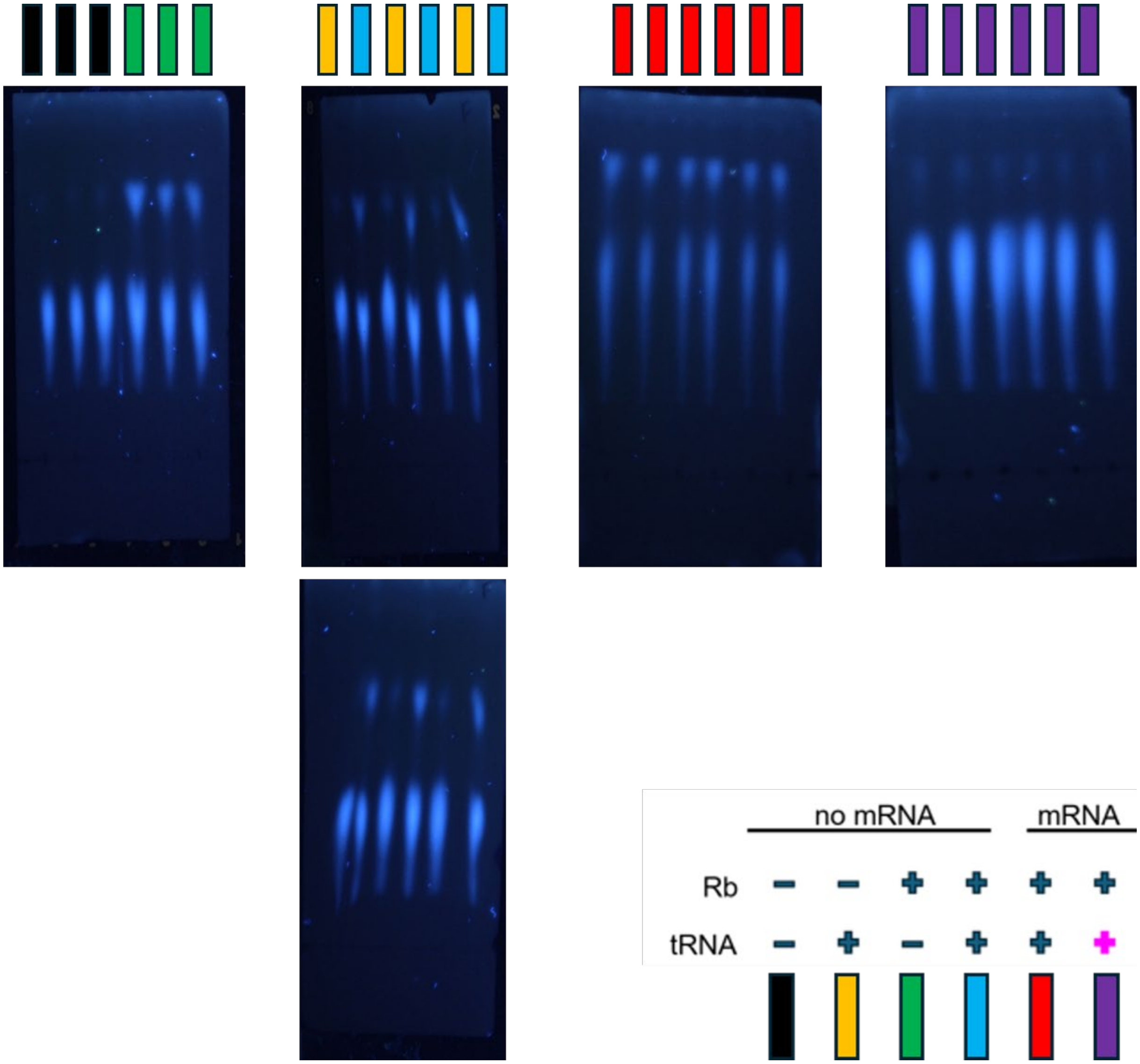

Figure S5. Original TLC images of D81F EF-Tu for Figure 1 in the main text. The experimental conditions are indicated by the colored bars in each column (bottom-right panel). If more than one triplicate is shown, they are displayed beneath the first set.

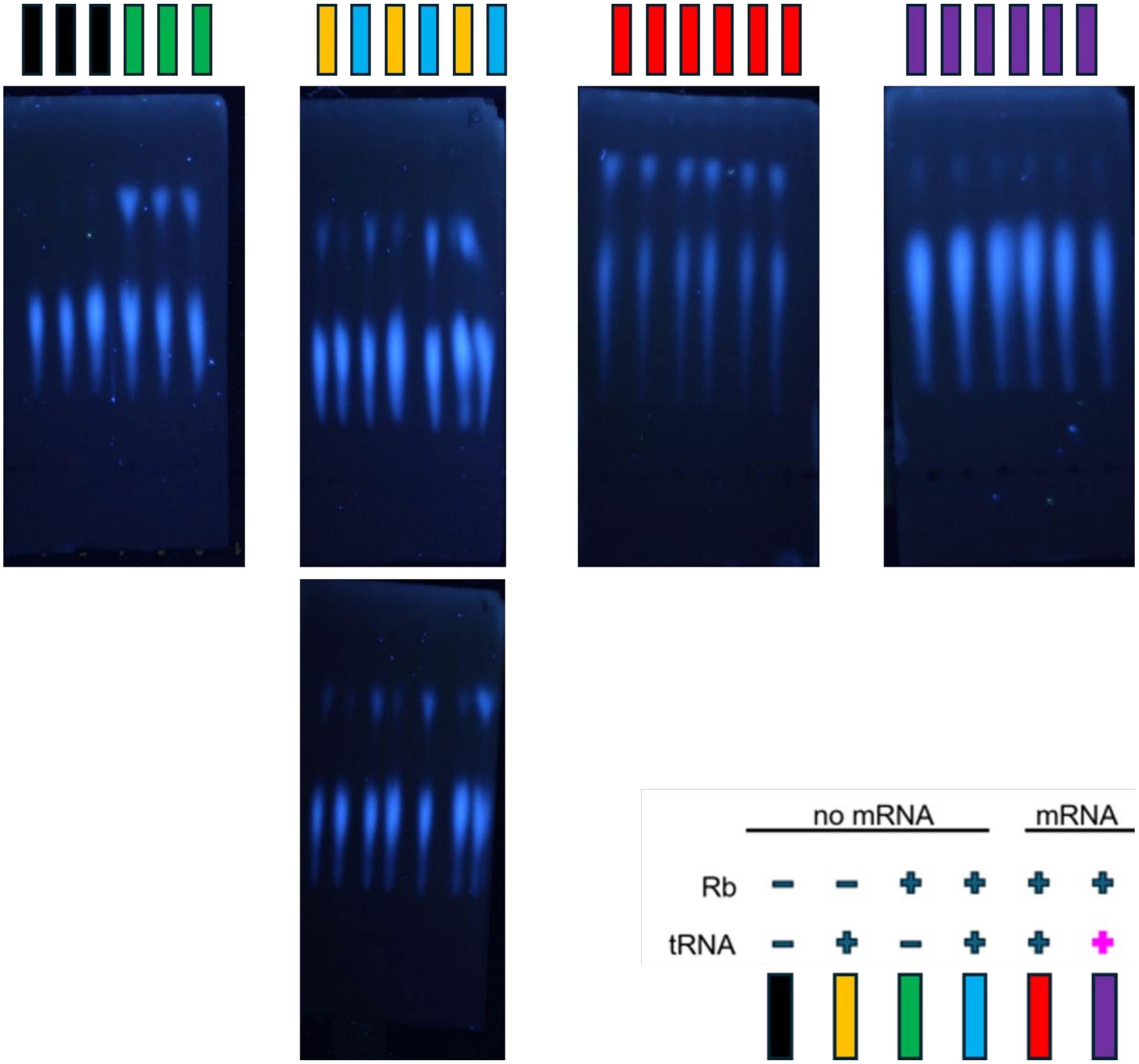

Figure S6. Original TLC images of D81K EF-Tu for Figure 1 in the main text. The experimental conditions are indicated by the colored bars in each column . If more than one triplicate is shown, they are displayed beneath the first set.

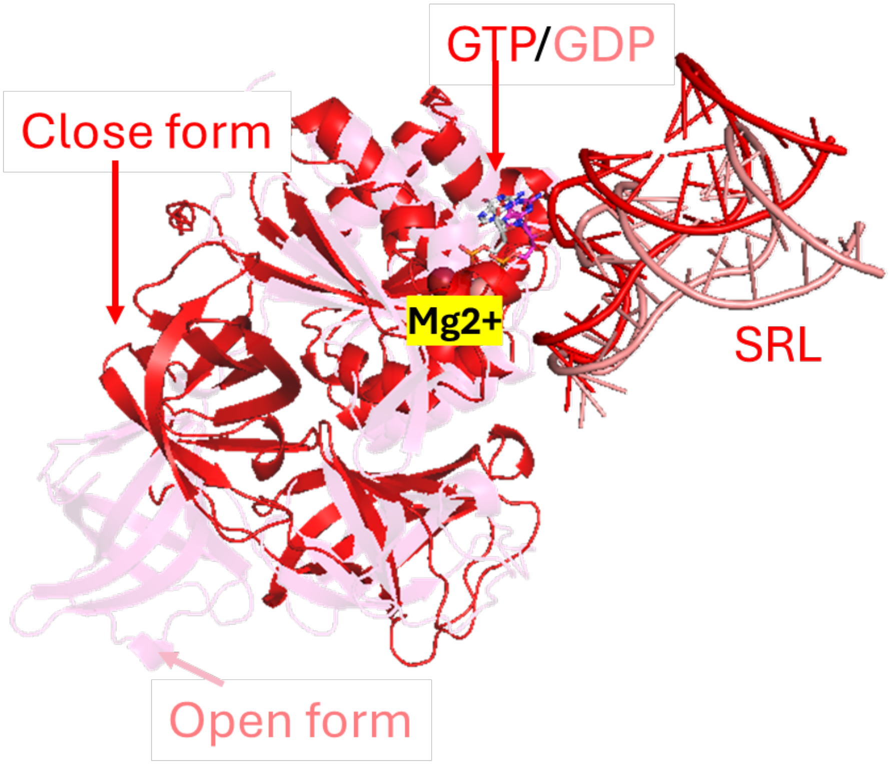

Figure S7. AlphaFold3 reproduces the experimentally observed closed and open conformations of WT EF-Tu, correlating with GTP- and GDP-bound states respectively.

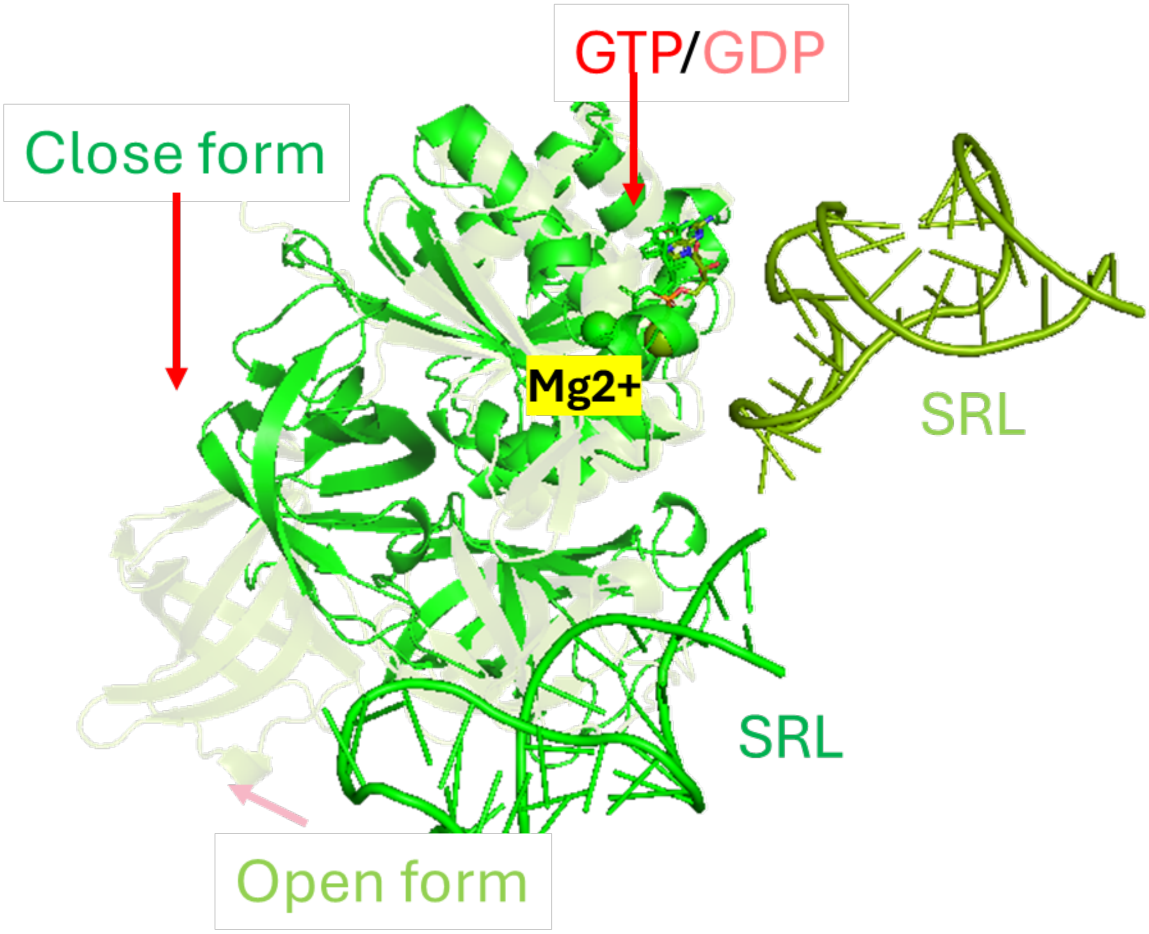

Figure S8. AlphaFold3 reproduces the experimentally observed closed and open conformations of D81A EF-Tu, correlating with GTP- and GDP-bound states respectively.

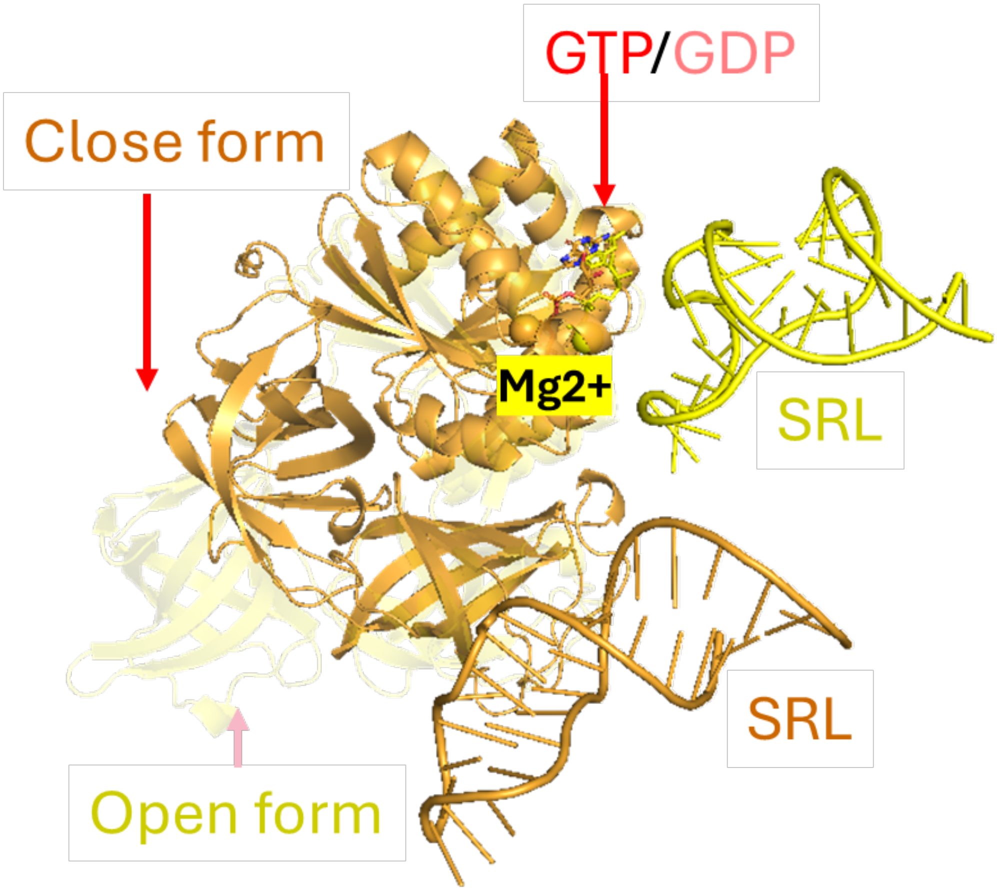

Figure S9. AlphaFold3 reproduces the experimentally observed closed and open conformations of D81F EF-Tu, correlating with GTP- and GDP-bound states respectively.

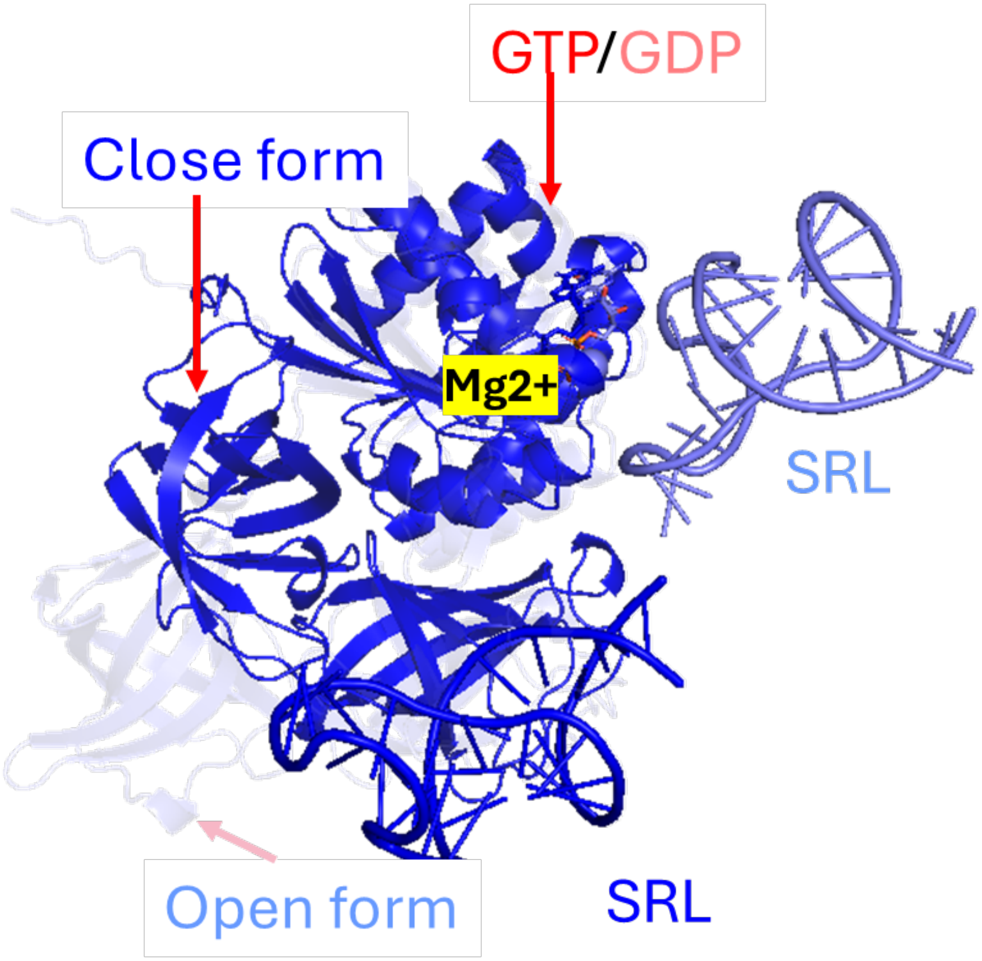

Figure S10. AlphaFold3 reproduces the experimentally observed closed and open conformations of D81K EF-Tu, correlating with GTP- and GDP-bound states respectively.

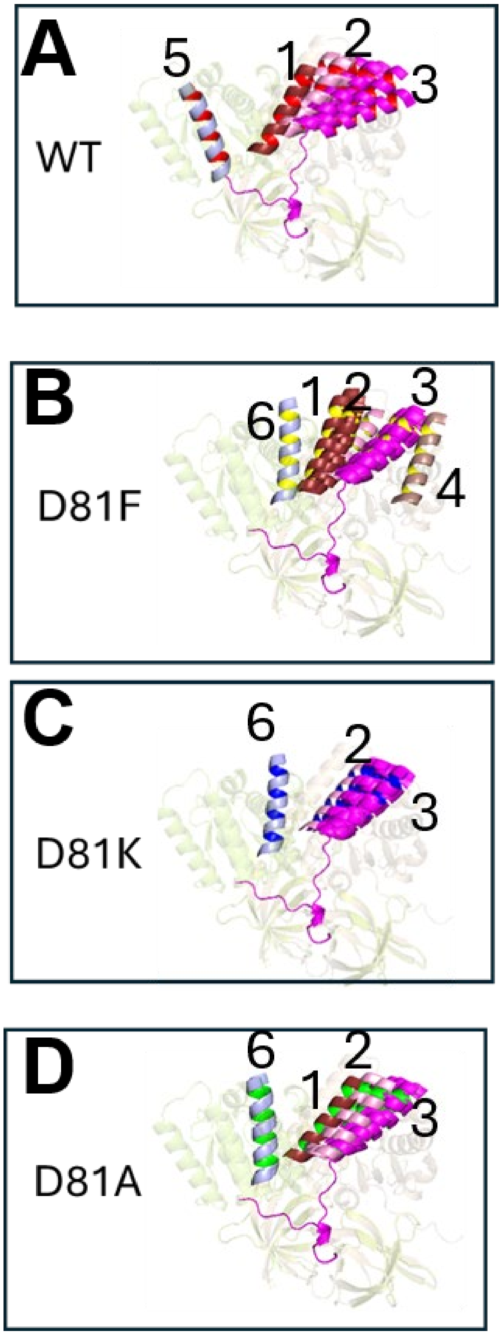

Figure S11. Conformational variation of domain I tracked by its terminal helix (residues 183–199), shown for WT (A), D81A (B), D81F (C), and D81K (D). Numbers in each panel indicate the conformational cluster assignments.

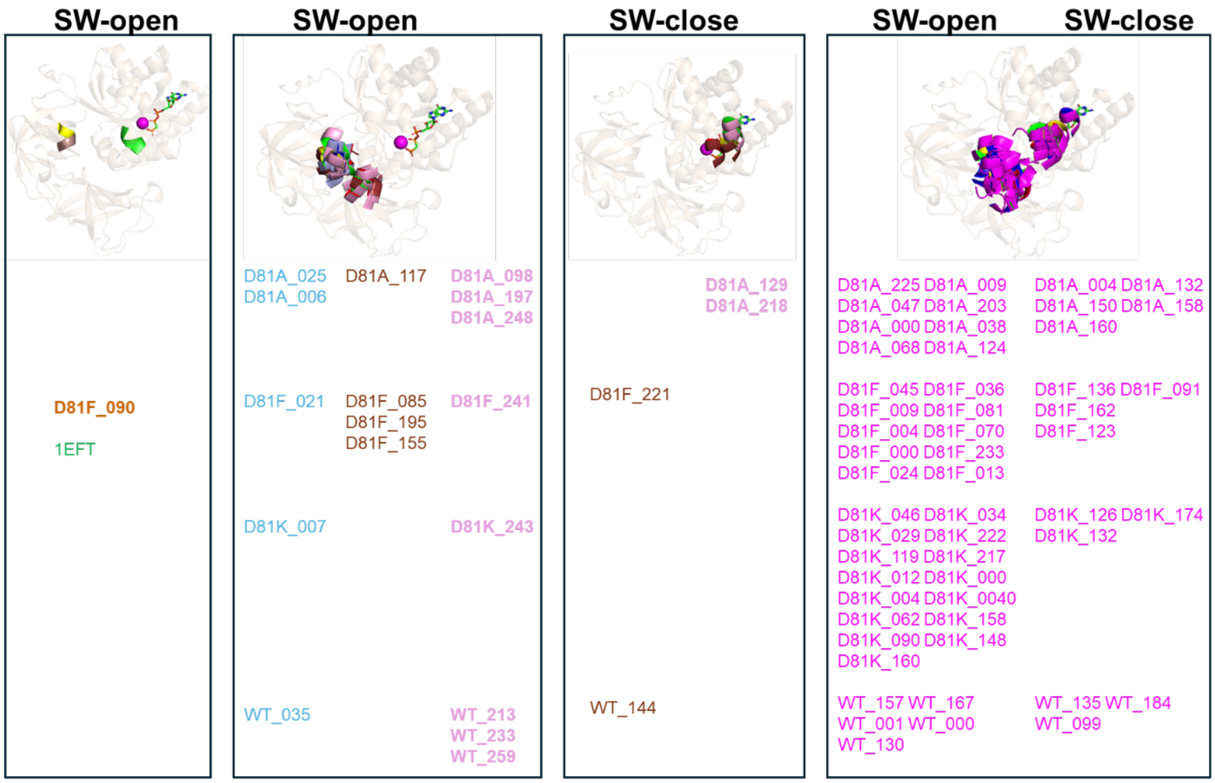

Figure S12. Conformations of the GTP binding pocket show no consistent correlation with global conformational clusters. Text colors match cluster assignments in Figure 3 of the main text. For example, D81F_085 and D81F_221 both adopt cluster 1 yet differ in GTP binding pocket state, demonstrating that global and local conformations are largely independent. This is most apparent in cluster 3 (magenta), where structures populate both open and closed pocket forms. However, clusters 5 and 6 (steel blue and light blue) show a tendency toward the switch-open form, and cluster 2 (pink) is predominantly switch-open with a minority in the switch-closed form, suggesting partial coupling between global and local conformational states in certain clusters. The EF-Tu scaffold is based on the D-close crystal structure (PDB: 1EFT). The purple sphere indicates Mg^{2+} and the chemical structure shown is GTP.

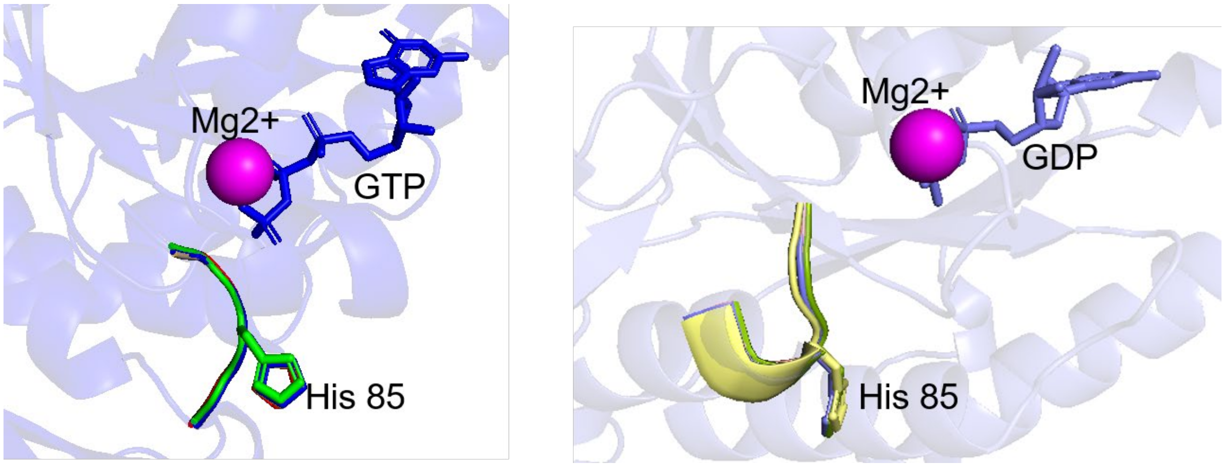

Figure S13. AlphaFold3-predicted His 84 position in EF-Tu complexes bound to GTP or GDP. Shown for WT and the D81A, D81F, and D81K mutants. His84 is positioned similarly across all variants in both nucleotide states. The Mg^{2+} ion and protein scaffold shown are from the D81K structures; the color scheme follows Figures S7–S10.

Table S1. Folding confidence metrices for EF-Tu (chain 0) with nucleotide (chain 1) and Mg^{2+} (chain 2). Iptm: interface confidence value between the chains; pae: position uncertainty between the chains. Iptim: overall interface confidence across all chains. Ptm: overall structural confidence.

Metric	d81a_gtp	d81f_gtp	d81k_gtp	wt_gtp	d81a_gdp	d81f_gdp	d81k_gdp	wt_gdp
chain_0	0.95	0.95	0.96	0.96	0.96	0.93	0.92	0.97
chain_1	0.7	0.69	0.76	0.75	0.72	0.65	0.64	0.75
chain_2	0.69	0.67	0.75	0.74	0.7	0.61	0.58	0.74
chain_0_1_iptm	0.97	0.97	0.97	0.97	0.98	0.97	0.98	0.98
chain_0_2_iptm	0.94	0.93	0.94	0.95	0.94	0.89	0.86	0.96
chain_1_2_iptm	0.44	0.41	0.55	0.52	0.46	0.34	0.31	0.53
chain_0_1_pae	0.83	0.86	0.85	0.83	0.83	0.87	0.83	0.82
chain_0_2_pae	1.74	1.83	1.6	1.45	1.81	2.9 [2]	3.7 [2]	1.37
chain_1_2_pae	2.45 [1]	2.64 [1]	1.98	1.95	2.18	3.58 [3]	3.48 [3]	1.64
iptm	0.97	0.96	0.97	0.97	0.97	0.97	0.97	0.98
ptm	0.83	0.82	0.81	0.82	0.91	0.84	0.89	0.91

[1]. position uncertainty between GTP-Mg^{2+} are higher in D81A and D81F.

[2]. position uncertainty between EF-Tu-Mg^{2+} are higher in D81F and D81K.

[3]. position uncertainty between GDP-Mg^{2+} are higher in D81F and D81K.

Table S2. Folding confidence metrices for EF-Tu (chain 0) with nucleotide (chain 1), Mg^{2+} (chain 2), 22-nt RNA (chain 3), and 30-nt SRL (chain 4). Iptm: interface confidence value between the chains; pae: position uncertainty between the chains. Iptim: overall interface confidence across all chains. Ptm: overall structural confidence.

Metric	a81_gtp	f81_gtp	k81_gtp	wt_gtp	a81_gdp	f81_gdp	k81_gdp	wt_gdp
chain_0	0.74	0.79	0.79	0.85	0.84	0.78	0.8	0.84
chain_1	0.48	0.49	0.51	0.59	0.48	0.46	0.47	0.5
chain_2	0.41	0.41	0.42	0.47	0.34	0.32	0.33	0.41
chain_3	0.28	0.3	0.28	0.27	0.26	0.2	0.22	0.25
chain_4	0.13	0.23	0.33	0.5	0.43	0.41	0.43	0.41
chain_0_1_iptm	0.97	0.97	0.97	0.97	0.97	0.97	0.97	0.97
chain_0_2_iptm	0.96	0.95	0.95	0.97	0.85	0.81	0.8	0.95
chain_0_3_iptm	0.68	0.72	0.6	0.6	0.71	0.55	0.61	0.66
chain_0_4_iptm	0.33	0.5	0.64	0.86	0.82	0.77	0.82	0.79
chain_1_2_iptm	0.56	0.52	0.53	0.55	0.27	0.25	0.25	0.4
chain_1_3_iptm	0.29	0.29	0.25	0.22	0.15	0.12	0.12	0.15
chain_1_4_iptm	0.09	0.19	0.29	0.61	0.51	0.52	0.53	0.48
chain_2_3_iptm	0.06	0.06	0.05	0.05	0.02	0.02	0.02	0.03
chain_2_4_iptm	0.04	0.09	0.16	0.32	0.21	0.21	0.23	0.24
chain_3_4_iptm	0.07	0.13	0.2	0.21	0.16	0.12	0.14	0.15
chain_0_1_pae	0.88	0.95	0.9	0.88	0.94	0.92	0.91	0.95
chain_0_2_pae	1.31	1.56	1.52	1.2	3.65[2]	4.62[2]	4.98[2]	1.55
chain_0_3_pae	4.54	4.11	6.4	6.87	3.71	7.71	4.83	5.11
chain_0_4_pae	14.17[1]	10.43[1]	6[1]	1.83	2.16	3.1	2.42	2.91
chain_1_2_pae	1.82	2	2.01	1.7	3.98[3]	4.54[3]	4.88[3]	2.18
chain_1_3_pae	5.65	5.41	7.94	7.59	7.47	11.73	9.22	8.6
chain_1_4_pae	15.77	13.78	9.71	1.98	2.53	3.24	2.7	2.77
chain_3_4_pae	16.16	13.38	10.81	11.25	9.62	14.17	12.34	10.88
iptm	0.77	0.76	0.78	0.84	0.82	0.77	0.8	0.8
ptm	0.83	0.81	0.83	0.84	0.87	0.83	0.84	0.85

[1]. position uncertainty between EF-Tu-SRL are higher in all mutants.

[2]. position uncertainty between EF-Tu-Mg^{2+} are higher in all mutants.

[3]. position uncertainty between GDP-Mg^{2+} are higher in all mutants.
